# Micro-vibration assisted dual-layer spiral microneedles to rapidly extract dermal interstitial fluid for minimally invasive detection of glucose

**DOI:** 10.1038/s41378-024-00850-x

**Published:** 2025-01-08

**Authors:** Khaled Mohammed Saifullah, Asim Mushtaq, Pouria Azarikhah, Philip D. Prewett, Graham J. Davies, Zahra Faraji Rad

**Affiliations:** 1 School of Engineering, University of Southern Queensland, Springfield, QLD 4300 Australia; 2 Centre for Future Materials, Institute for Advanced Engineering and Space Sciences, University of Southern Queensland, Springfield, QLD Australia; 3 School of Engineering, University of Birmingham, Birmingham, B15 2TT UK; 4 Oxacus Ltd, Dorchester-on-Thames, OX10 7HN UK; 5 Faculty of Engineering, UNSW Australia, Sydney, NSW 2052 Australia; 6 College of Engineering & Physical Sciences, School of Engineering, University of Birmingham, Birmingham, B15 2TT UK

**Keywords:** Engineering, Materials science

## Abstract

Various hydrogels have been explored to create minimally invasive microneedles (MNs) to extract interstitial fluid (ISF). However, current methods are time-consuming and typically require 10–15 min to extract 3–5 mg of ISF. This study introduces two spiral-shaped swellable MN arrays: one made of gelatin methacryloyl (GelMA) and polyvinyl alcohol (PVA), and the other incorporating a combination of PVA, polyvinylpyrrolidone (PVP), and hyaluronic acid (HA) for fast ISF extraction. These MN arrays demonstrated a rapid swelling ratio of 560 ± 79.6% and 370 ± 34.1% in artificial ISF within 10 min, respectively. Additionally, this study proposes a novel method that combines MNs with a custom-designed Arduino-based applicator vibrating at frequency ranges (50–100 Hz) to improve skin penetration efficiency, thereby enhancing the uptake of ISF in ex vivo. This dynamic combination enables GelMA/PVA MNs to rapidly uptake 6.41 ± 1.01 mg of ISF in just 5 min, while PVA/PVP/HA MNs extract 5.38 ± 0.77 mg of ISF within the same timeframe. To validate the capability of the MNs to recover glucose as the target biomarker, a mild heating procedure is used, followed by determining glucose concentration using a d-glucose content assay kit. The efficient extraction of ISF and glucose detection capabilities of the spiral MNs suggest their potential for rapid and minimally invasive biomarker sensing.

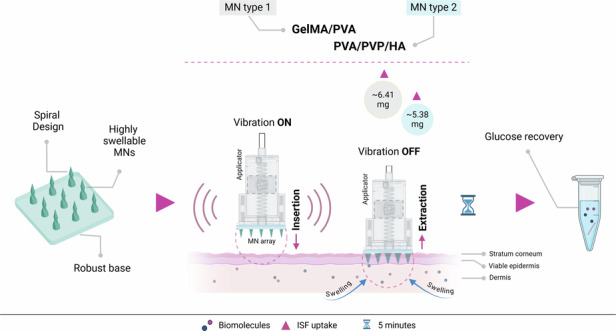

## Introduction

The need for minimally invasive techniques has led to significant advancements in body fluid extraction and its correlation with blood sampling and point-of-care diagnostics^1^. While a blood test is the gold standard in diagnostics for assessing various health parameters and detecting various medical conditions, it presents challenges such as invasiveness and infection risks. It may also be unavailable in resource-limited settings^2–6^. Additionally, the potential of other body fluids like saliva, urine and sweat is limited by minimal to absent biomarker concentrations^7^.

ISF is a colorless, water-like fluid that resides in the spaces between blood vessels and cells^8,9^. It plays a crucial role in the nutrient transport system, carrying analytes, nutrients, and waste products away from the blood capillaries through diffusion^10^. Plasma in blood contains clotting factors that initiate the formation of a clot when an injury occurs, preventing excessive bleeding. Serum is essentially plasma without these clotting factors and is derived from blood plasma by allowing the blood to clot and then removing the resultant clot^11^. Unlike plasma and serum, ISF does not contain any clotting factors, and due to this, it does not clot^12^. This is an important characteristic because clotting can often complicate the analysis and use of biological samples. The absence of clotting in ISF presents a unique advantage, making it a viable alternative to blood or other biological samples in real-time diagnostics^13^. Traditional techniques to extract the ISF, such as suction blister and sonophoresis, can lead to significant disruption or alteration in the integrity and structure of the outermost layer of the skin, known as the stratum corneum (SC) and cause local trauma to the skin^14–16^.

To address this issue, MNs have emerged as a prominent tool to harness the ISF for biomarker detection due to their unique properties and advantages^17^. MNs are tiny, needle-like devices that can penetrate the skin to reach the ISF without causing significant discomfort. This is a stark contrast to conventional hypodermic needles, which can be painful and invasive^18^. Different types of MNs have been studied, such as solid, coated, dissolvable, swellable, and hollow, made from silicon, metals, polymers, and sugar for drug delivery and fluid extraction^19^. However, swellable MNs, a type of solid MNs, offer a unique advantage in ISF extraction. They are made from swellable polymers that absorb ISF upon insertion into the skin and swell. This property allows for a minimally invasive extraction of ISF and storage within the MN themselves^20^.

Cross-linked hydrogels such as PVA/PVP and GelMA have demonstrated a good swelling ratio, a crucial factor for efficient ISF extraction^21–24^. PVA has been extensively used in MNs due to its excellent biocompatibility and swelling properties^25^. Combining PVP with PVA can also improve the mechanical properties of the hydrogel^23,25^. GelMA hydrogels have attracted significant attention in the biomedical field due to their tunable mechanical properties that can be adjusted by varying factors such as ultraviolet (UV) polymerization conditions and concentrations^24^. However, despite these promising characteristics, the application of these hydrogels is primarily limited, mainly due to the low volume of ISF extracted and the prolonged extraction time. For example, Zhu et al. developed GelMA-based MNs that could extract only 2.5 mg of ISF in 10 min^26^. Xu et al. reported that PVA/PVP-based MNs cross-linked with glutardialdehyde could extract 4.4 mg of ISF in 12 min^27^. Fonseca et al. developed cross-linked c-GelMA MNs to extract and detect urea levels in ISF. The c-GelMA hydrogel MNs extracted 3.5 ± 0.1 mg of ISF from the agarose gel tests in 30 min^28^. Chang et al. also tested swellable MNs composed of methacrylated hyaluronic acid that extracted 2.3 ± 0.4 mg of ISF from female nude mice in 10 min^29^.

Therefore, there is a need for rapid ISF extraction and biomarker analysis. This study introduces two distinct types of cross-linked, dual-layer spiral structured MNs made of GelMA/PVA and PVA/PVP/HA hydrogels for rapid ISF extraction. To the author’s knowledge, the spiral-shaped MN configuration represents a novel design that has not been explored previously. The chosen hydrogel combinations (GelMA/PVA and PVA/PVP/HA) significantly enhanced the swelling behavior of the MNs compared to previous studies^26–28^. This study further analyzes the importance of a uniform base layer for effective MN penetration into the skin. Due to its flexibility, Polyethylene Glycol Diacrylate (PEGDA) is selected for the GelMA/PVA MN array base, while PVP is chosen for the PVA/PVP/HA MN array base. Optimal drying times for the PVP base are determined through a two-step approach, with GelMA MNs requiring a shorter drying time. This study further investigates the swelling and mechanical properties of the MNs to assess their capabilities in insertion into the skin and ISF extraction. Swelling tests are conducted in both PBS and artificial ISF, while compression tests are utilized to assess the mechanical characteristics of the MNs.

**Fig. 1:**
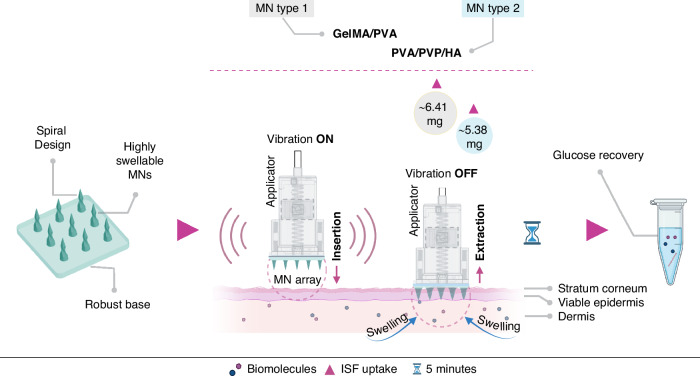
Schematic illustration of minimally invasive ISF extraction and glucose recovery using highly swellable spiral MNs with the custom-made applicator to generate micro-vibration during the insertion phase

The study also introduces an advanced version of our patented MN applicator capable of inserting MNs at a velocity of 4.5 m/s with vibration (50–100 Hz), developed by our group for the first time. The updated version of the applicator allows precise control over vibration during and immediately after insertion using two eccentric rotating mass (ERM) micro-vibration motors that can easily be controlled independently or in unison through the Arduino IDE. This supports the feasibility of micro-vibration-assisted ISF extraction, a novel technique developed in this study. The results indicated that in conjunction with various other factors (e.g., MN design, material properties, swelling behavior of the MN material), vibration-assisted insertion significantly improves penetration effects and enables more ISF extraction. Using this approach, GelMA/PVA and PVA/PVP/HA MN arrays are tested on porcine ear skin, extracting 6.41 ± 1.01 mg and 5.38 ± 0.77 mg of ISF in just 5 min. Furthermore, the successful recovery of various glucose concentrations is validated through in vitro agarose gel experiments using the GelMA/PVA and PVA/PVP/HA MN arrays. A schematic of minimally invasive rapid extraction of ISF and recovery of glucose using micro-vibration-assisted spiral MNs is presented in Fig. 1.

## Results and discussion

### Spiral design

MNs were designed in a spiral pattern by incorporating two squares of 0.15 mm and 0.20 mm in the structure. The distance between the squares was 0.4 mm, and the tip and base of the needle were constructed as circles. MN initial design had a height of 1 mm, a tip diameter of 0.02 mm, a base diameter of 0.3 mm and the distance between the needles was set at 0.40 mm. The primary objective of adopting a spiral design was to provide interlocking capabilities for effective ISF extraction. This could be due to the increased number of vertices in the spiral design, which could aid in achieving a more stable penetration. This stability could reduce the likelihood of MN displacement during use. Moreover, the spiral form offers an increased surface area in contact with the skin compared to traditional conical shapes. This could potentially enhance the efficiency of ISF extraction by providing a larger surface for the fluid to adhere to and be drawn out during the retraction of the MNs. In the current study, the MNs were fabricated with an anticlockwise twist, fixed upon insertion and did not allow rotation. The number of MNs per array was 15 × 15 on a base plate of 12 mm × 12 mm. The thickness of the MN array base was set to 1.25 mm, which provided sufficient support during the PDMS mold replication for the MN solution to be added without any additional steps required^30^.

### Fabrication of spiral MNs and optimization of base layers

This study used projection micro stereolithography (PμSL) to print 15 × 15 master MN arrays over a 12 mm × 12 mm area. This advanced technology enables 3D printing of miniaturized parts with 2 μm resolution and +/− 10 μm accuracy at scale. PμSL represents the link of two major technology trends: 3D printing and miniaturization. However, miniaturization is limited by the difficulty in prototyping and cost-effective manufacturing of the devices. PμSL is a stereolithography technique incorporating a digital light processing engine, precision optics, motion control, and advanced software. Stereolithography produces parts in layers using a photochemical process. A photosensitive liquid resin is exposed to light; consequently, polymeric cross-linking and solidification occur. A flash of UV light, 405 nm, causes the rapid photo-polymerization of an entire resin layer. Like other 3D printing processes, it uses a CAD file as an input and is then sliced into a series of 2D images called digital masks that show or hide specific areas of a layer. Each layer has a mask, and each layer is added until the entire 3D structure is complete. Slicing data was sent to the 3D printing system to fabricate individual layers. PμSL technology can achieve resolutions of several micrometers or hundreds of nanometers by controlling the projection lens. The master MN array was printed in high temperature resistance (HTL) material using this advanced technology.

**Fig. 2:**
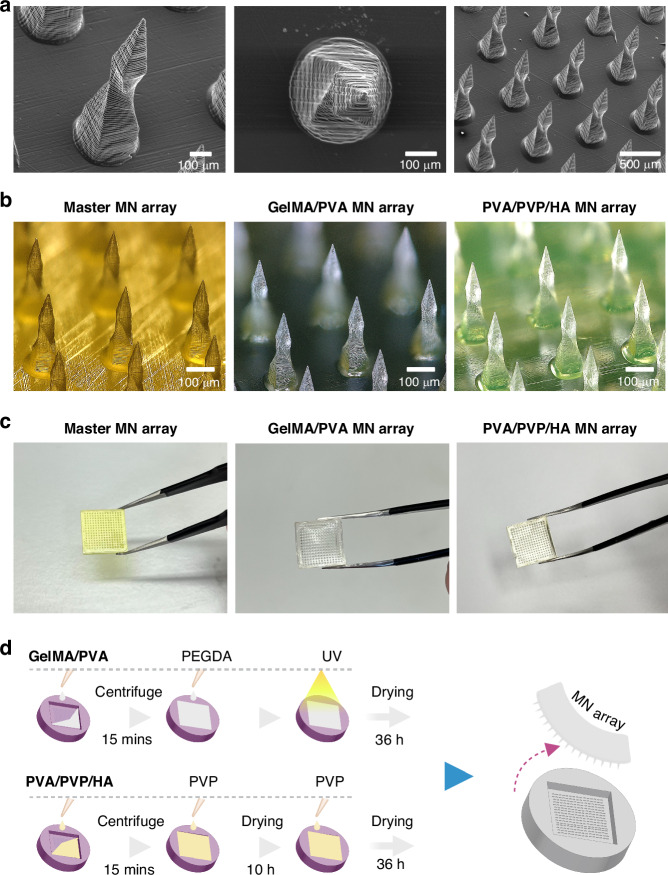
Design and fabrication process of the spiral-shaped GelMA/PVA and PVA/PVP/HA MNs. **a** SEM images showing the complex spiral structure of the MN (left), tip view (middle) and an array where MNs are distributed uniformly across the base (right). **b** Optical images (45° tilt) of the master MNs 3D printed (left), GelMA/PVA (20/3, w/v) MN array with the base layer (middle) and 12/5/1, w/v) MN array with the base layer (right) in differential interference contrast mode. **c** The captured images of the master MN array (left), GelMA/PVA (20/3, w/v) array (middle) and PVA/PVP/HA MN (12/5/1, w/v) array (right) containing 225 MNs in each array. **d** Schematic representation of the fabrication process for GelMA/PVA and PVA/PVP/HA MN arrays, showing centrifugation, UV curing, and drying steps leading to the final MN array

After creating the master MN, it was used to create PDMS negative molds (Supplementary Fig. S1) to replicate GelMA/PVA and PVA/PVP/HA MN arrays. All MN replicas were formed without any defect, resembling the spiral shape of the master array, as shown in Fig. 2a; however, longitudinal shrinkage was observed across all MN types (master and replica) compared to their initial CAD dimension (1000 µm). The 3D printed master MNs shrank by ~29.5% (705 ± 2.12 µm) longitudinally, which could be due to the 3D printing characteristics such as print speed and material exposure time during the print that can unevenly distort and lead to a higher overall shrinkage. The GelMA/PVA and PVA/PVP/HA MNs also showed shrinkage in height of ~32.6% (674 ± 4.97 µm) and ~31.4% (685 ± 7.45 µm) to the original CAD dimension, respectively. Compared to the 3D printed master MNs, these two types of MNs’ shrinkage were significantly lower at ~3.1% and ~1.9%, respectively. The master MNs had a sharp tip with a diameter of 4.47 ± 0.32 µm, whereas GelMA/PVA and PVA/PVP/HA MNs had a tip diameter of 7.12 ± 0.91 µm and 5.6 ± 0.35 µm, respectively. These are well below 15 µm, which is fundamental for successful insertion into the skin^31^. This suggests the successful fabrication of polydimethylsiloxane (PDMS) molds and precise replication of the spiral geometry. In Fig. 2b, c, optical and captured images of MNs show consistent structural integrity among master, GelMA/PVA, and PVA/PVP/HA MNs. The spiral shape, as well as the viability and integrity of the tips, remains identical across these variants.

For precise insertion and ease of attachment of the MN arrays to the applicator, the base layer of the MN array should be reasonably flat and uniform, particularly for larger array sizes (≥12 mm × 12 mm). Lack of uniformity in the base may lead to insufficient penetration and effectiveness. For GelMA/PVA MNs, PEGDA was chosen as the base layer due to its flexible mechanical properties, which can be tuned by adjusting the amount of Irgacure and UV exposure time^32^. This shared component (GelMA/PVA MNs and PEGDA base) facilitated a strong bond, forming a cohesive, single-layer structure in the final MN array. However, the attempt to incorporate PEGDA into PVA/PVP/HA solutions as a base did not work as expected. For instance, PEGDA did not form a uniform layer with the PVA/PVP/HA solution. Due to its photoinitiator content, the PEGDA base hardened more rapidly when exposed to UV light than the PVA/PVP/HA solution, leading to inadequate bonding between the two layers. To address this, three tests were conducted: one with the combination of PVA/PVP (30/30, w/v) and the other two with PVP (30%, w/v) and PVA (30%, w/v). Uniformity was observed in all formulations, but the duration of solution drying varied proportionally to the concentration of PVA or PVP. Considering that MNs would be dissolved in the buffer solution to recover the biomarker, it was challenging to dissolve PVA/PVP (30%, 30%, w/v). On the other hand, preparing PVA (30%, w/v) with a higher molecular weight (89 kDa) proved to be a time-consuming process due to its exceptionally high viscosity. Therefore, PVP (30%, w/v) was considered the preferred solution for ease of preparation, lower viscosity, convenience for pipetting, and adequate bonding with PVA/PVP/HA MNs.

The drying time for the PVP base was optimized using a two-step strategy. Initially, 200 µL of the PVA/PVP/HA solution was added into the molds and centrifuged to form the MNs, followed by an immediate overfill of PVP to form the base and drying for 10 h (Supplementary Fig. S2). Additional PVP was added and left to dry for another 24 h to develop a uniform MN base structure. Figure 2d illustrates the optimization process of the MN arrays and their base layer. It was noted that large variations in the optimal drying time resulted in uneven drying, with shrinkage occurring in the center while the corners remained rigid. GelMA MNs were dried for 36 h, after which they were successfully peeled off without further issues.

### Swelling kinetics and PBS uptake in vitro

Swelling properties of the material play a pivotal role in determining the efficiency of swellable MNs in extracting ISF. MNs with enhanced swelling ratio are more likely to rapidly extract a higher volume of ISF within a shorter period (<5 min), which would benefit users by reducing the time required for ISF sampling and increasing the accuracy of diagnostic tests.

**Fig. 3:**
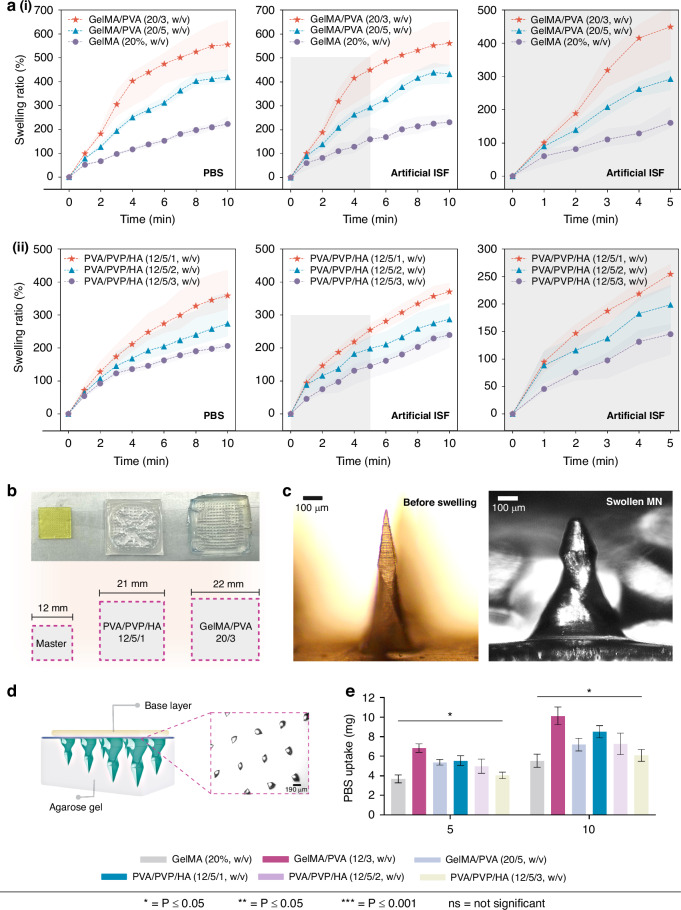
Swelling behavior, morphology, and PBS uptake of GelMA/PVA and PVA/PVP/HA MNs. **a** (i) Swelling rate of the GelMA/PVA MNs in PBS buffer (left) and artificial ISF (middle) at 37 °C with the first 5 min in artificial ISF highlighted (right). **a** (ii) Swelling rate of the PVA/PVP/HA MNs in PBS buffer (left) and artificial ISF (middle) at 37 °C with the first 5 min in artificial ISF highlighted (right). **b** A visual comparison of the MN arrays shows their increased size compared to the original master template. **c** Microscopic images of a single MN showing morphological changes before and after swelling in PBS buffer. **d** Schematic illustration of MNs penetration into agarose gel (3%, w/v) for in vitro swelling test. The micro-holes left by the MNs are shown in the closed-up section. **e** PBS uptake (mg) by the MNs after 5 and 10 min of swelling in agarose gel (3%, w/v)

The swelling ratios of the MNs were conducted in artificial ISF and PBS (Phosphate-buffered Saline) for up to 10 min. The results in Fig. 3a(i-ii) demonstrated that the swelling ability of the MNs increases over time after immersion. In artificial ISF, GelMA (20%, w/v) alone had a relatively lower swelling ratio of 230 ± 20.8%. However, the incorporation of 3% (w/v) and 5% (w/v) PVA increased the swelling to 560 ± 79.6% and 432 ± 35%, respectively, which are higher than similar reported studies^26,28^. This enhanced swelling can be attributed to increased hydrophilic sites within the GelMA matrix. These results showed that increasing PVA concentration from 3% (w/v) to 5% (w/v) did not result in a linear improvement in swelling and formed a denser structure with reduced porosity. Therefore, this observation suggests that further increasing the PVA concentration will unlikely enhance the swelling of the GelMA/PVA MNs.

Further experiments were conducted with varying concentrations of PVA and PVP only. However, the obtained swelling ability of the MNs was not optimal (Supplementary Fig. S3). Therefore, HA was introduced, and three different concentrations of HA (1%, 2%, 3%, w/v) were prepared while maintaining the PVA and PVP concentrations at 12% (w/v) and 5% (w/v), respectively. The variations in swelling ratios can be related to the different concentrations of HA. Introducing 1% (w/v) HA resulted in the highest swelling ratio of 370 ± 18.5%, indicating a direct influence to absorb the artificial ISF and remain intact. The increased swelling is likely due to the larger pore size of HA molecules, which allowed for greater ISF uptake. As the HA concentration increased to 2% (w/v), a slightly lower swelling ratio of 286 ± 48% was observed. Notably, with a 3% (w/v) HA concentration, the swelling ratio further decreased to 239 ± 21.3%. Therefore, incorporating small amounts of HA can enhance the swelling behavior of the PVA/PVP hydrogel, but excessive HA could occupy a significant portion of the hydrogel network. This limits the capacity of the hydrogel MNs to absorb and retain fluid effectively.

Similar experiments were carried out in PBS for the swelling test. The MNs exhibited subtle differences in PBS compared to artificial ISF. For example, GelMA/PVA compositions showed a slightly higher swelling tendency when immersed in artificial ISF, 560 ± 79.6% compared to 555 ± 89.8% in PBS, possibly due to specific interactions with certain constituents present in ISF. PVA/PVP/HA compositions also exhibited lower swelling in PBS, 358 ± 45.4%, compared to 370 ± 18.5% in artificial ISF. This variation in swelling characteristics between the two different environments (artificial ISF and PBS) could be attributed to differences in the chemical composition and concentrations of the solvents. Although Swelling ability can be influenced by various factors^33^ (e.g., chemical composition of the hydrogel network and properties of the surrounding environment), the result suggested that the swelling ratio of these MNs is relatively consistent between PBS and artificial ISF.

Expansion and swelling of the MN arrays were also very noticeable visually. As the MNs swelled, they significantly increased in size, confirming their ability to effectively absorb PBS. The results indicated that the GelMA/PVA (20/3, w/v) MN array exhibited a substantial increase in size, swelling to 1.7 times its original dimensions (master MN array). Similarly, the PVA/PVP/HA (12/5/1, w/v) MNs also demonstrated significant swelling, expanding to 1.8 times their initial size (master MN array). Both types of MNs showed exceptional ability to absorb PBS and increase in volume in 5 min, as shown in Fig. 3b, with the PVA/PVP/HA (12/5/1, w/v) MN array being slightly smaller in size compared to the GelMA/PVA (20/3, w/v) MN array. Figure 3c shows the image of a MN before and after 5 min of swelling, which caused the MN to expand, resulting in a broader and less defined tip than its original shape.

Experiments were also conducted on the skin model constructed using agarose hydrogel to assess the PBS uptake capacity of MNs, as illustrated in Fig. 3d. A consistent pattern of increased PBS uptake was observed across various GelMA/PVA and PVA/PVP/HA compositions, as shown in Fig. 3e. Among them, GelMA/PVA (20/3, w/v) exhibited the highest PBS uptake of 10.12 ± 0.9 mg, followed by 7.2 ± 0.64 mg and 5.5 ± 0.66 mg for GelMA/PVA (20/5, w/v) and GelMA (20%, w/v), respectively. These results are higher than those reported in previous studies and align with the observed trend in swelling behavior^26,28,34^. Based on the experimental results, GelMA/PVA (20/3, w/v) appeared most suitable for swellable MNs among similar compositions. The extracted amount of PVA/PVP/HA MNs varied depending on the ratio of PVP/PVP to HA. The highest PBS volume was obtained with PVA/PVP/HA (12/5/1, w/v), extracting 8.5 ± 0.62 mg from the agarose gel. Other compositions, such as PVA/PVP/HA (12/5/2, w/v) and PVA/PVP/HA (12/5/3, w/v), extracted 7.2 ± 1.1 mg and 6.08 ± 0.59 mg, respectively. These values also exceeded those reported in similar studies^27,33,35^. Additionally, the PBS uptake ability of these MNs aligns with the swelling trend obtained earlier. Therefore, PVA/PVP/HA (12/5/1, w/v) was chosen as the preferred solution for further assessments. A summary of all swelling test results on both PBS and artificial ISF at 37 °C is provided in Supplementary Table S1.

### Mechanical properties of the spiral MNs

**Fig. 4:**
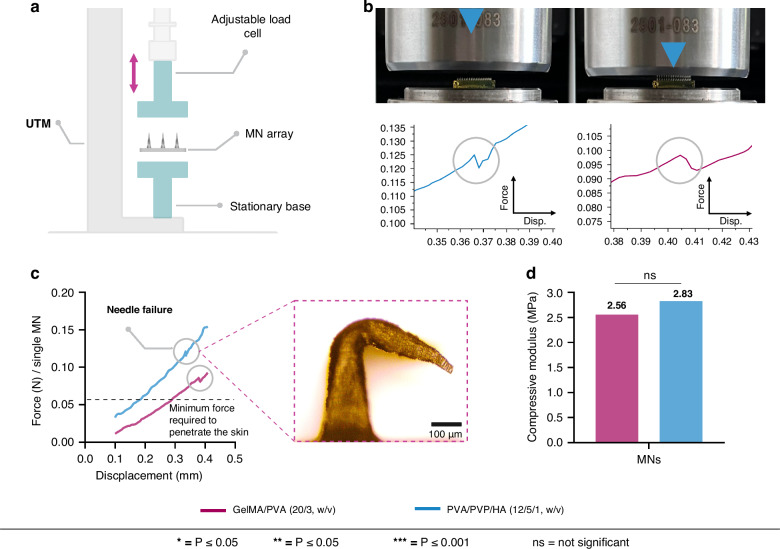
Mechanical testing of the GelMA/PVA and PVA/PVP/HA MNs. **a** Schematic illustration shows the setup of the UTM used for the compressive load test. The MN array is placed between a stationary base and an adjustable load cell that applies force while the force-displacement data is recorded. **b** The testing setup with the MN arrays under the load cell. The force-displacement curves for each type of MN (PVA/PVP/HA on the left and GelMA/PVA on the right) show the point at which the MNs reach their fracture force, with a noticeable peak followed by a drop indicating needle failure. **c** Comparison of the force-displacement curves of a single MN between GelMA/PVA (20/3, w/v) and PVA/PVP/HA (12/5/1, w/v) compositions showing the needle failure points, peaks, and the dotted line represents the minimum force required to penetrate the skin (0.058 N). Both MN types surpass this threshold, with PVA/PVP/HA showing a higher fracture force. The close-up section on the right shows the deformation of the MN during the compression test. **d** The Bar diagram shows the compressive modulus of the two types of MNs

Although skin penetration force can be influenced by age, gender, and race, studies have reported that a minimum fracture force of 0.058 N per needle is necessary to penetrate the skin successfully^36,37^. This represents that a single MN must overcome the required fracture force to pierce through the outermost layer (SC), which is the primary barrier to penetration. Efficient extraction of ISF also requires MNs to withstand the skin barrier, SC, without breaking. Therefore, a compressive load test was conducted to assess the structural integrity of the spiral MNs. MN array (15 × 15) was placed between the plates of a universal testing machine (UTM) to record the force-displacement curve, as illustrated in Fig. 4a. Although each array in the test had 225 MNs, the force measurement was calculated to represent a single needle in the force-displacement curves which provides critical insights into the mechanical behavior of the MNs. When the tip of the MN first contacts the upper plate of the UTM, the force increases to a peak, then drops to a low point before rising to a second peak. The initial peak indicates the failure load, resulting in permanent deformation of the MN structure. Figure 4b shows the impact of compression (0.5 mm/min) on the MN structure and their corresponding peaks as both types of MNs deform in the vertical direction.

For GelMA/PVA (20/3, w/v), the compressive load applied to each MN was gradually increased from 0 to 0.18 N as the displacement reached 0.5 mm. The fracture force of the single MN was 0.11 N, which occurred at a displacement of 0.37 mm. For the PVA/PVP/HA (12/5/1, w/v) MNs, the compressive load reached 0.19 N by the end of the test, and the measured fracture force for a single MN was 0.13 N. This suggests that the mechanical strength of each PVA/PVP/HA (12/5/1, w/v) MN was ~18% higher than that of the GelMA/PVA (20/3, w/v) MNs, as indicated by their respective fracture forces (0.13 N compared to 0.11 N). Both fracture forces of the GelMA/PVA and PVA/PVP/HA MNs were significantly higher than the minimum force required (0.058 N) to penetrate human skin. Specifically, the fracture force overcome by a single GelMA/PVA (20/3, w/v) MN was ~1.8 times higher than the required threshold, while that of each PVA/PVP/HA (12/5/1, w/v) MN was ~2.2 times higher. These fracture forces are significantly higher than the required force (0.058 N/needle) to penetrate human skin, as highlighted (dotted grey line) in Fig. 4c. Additionally, the compressive modulus for GelMA/PVA (20/3, w/v) was 2.56 MPa when the GelMA concentration remained fixed at 20% (w/v) with a cross-linking time of 250 sec. Meanwhile, the compressive modulus of PVA/PVP/HA (12/5/1, w/v) was 2.83 MPa when the Irgacure concentration was 0.5% (w/v). Figure 4d shows the differences in the respective compressive modulus values of the two MN types.

Many studies of MNs have demonstrated good mechanical properties using similar materials such as PVA/PVA, GelMA, and HA. For example, Zhu et al., reported that compressive modulus increased from 3.35 ± 0.62 MPa to 7.21 ± 1.74 MPa with the variation of GelMA concentration from 10% (m/v) to 25% (m/v)^26^. Khatabi et al. reported MNs made of PVA/HA with a failure load of 0.59 N, ten times higher than the required force to penetrate the skin for the application of drug delivery^38^. Xu et al., studied swellable PVA/PVP MNs by changing the PVA concentration that can uptake ISF without significant deformation of the MN tip^27^. The GelMA/PVA (20/3, w/v) and PVA/PVP/HA (12/5/1, w/v) MNs reported in this study demonstrated robust mechanical properties compared to similar studies. It is important to note that MN strength can impact the ability to uptake ISF. Swellable MNs with sufficient strength to penetrate the skin can enhance the extraction volume of ISF. Increasing MN stiffness, however, reduces the size of pores within the structure and prolongs the time needed to uptake ISF. Therefore, a balance between mechanical strength and porosity must be considered during swellable MN array design and development.

### Surface characterization

The surface properties of MNs are also crucial for their effectiveness in skin penetration, ISF extraction, and comfort during use. The roughness of MNs, referring to the micro-scale variations in height across the surface, can influence interactions with the skin^39^. A rough surface may hinder smooth penetration, requiring more force for skin penetration, which can increase pain sensitivity and potentially cause damage to both MNs and the skin.

**Fig. 5:**
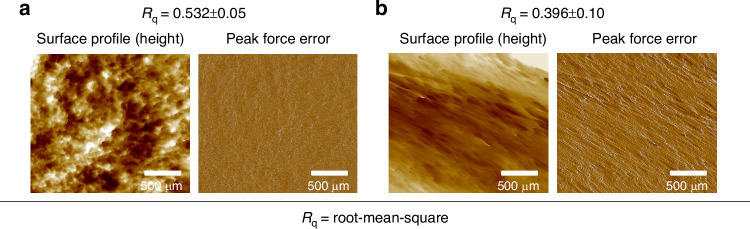
AFM images showing the surface topography of MNs. Surface profile with visible height variations and corrected peak force error for variations in tip-sample interactions of the **a** GelMA/PVA (20/3, w/v) **b** PVA/PVP/HA (12/5/1, w/v). AFM scan size: 500 nm × 500 nm

MNs were assessed for surface roughness properties using Atomic Force Microscopy (AFM). The GelMA/PVA (20/3, w/v) MNs exhibited a roughness of 0.532 ± 0.05 µm, while the PVA/PVP/HA (12/5/1, w/v) MNs showed a lower roughness value of 0.396 ± 0.10 µm without any additional coatings, as shown in Fig. 5a, b. Since GelMA/PVA (20/3, w/v) is composed of different materials and had higher swelling (a more porous structure), this likely contributed to a ~ 34.34% increase in surface roughness compared to the PVA/PVP/HA (12/5/1, w/v) MNs. It was also notable that GelMA/PVA (20/3, w/v) appears to have more porous and variable topography, while PVA/PVP/HA (12/5/1, w/v) appears smoother with less height variation. Analyzing the roughness of the surface provides insightful details about MNs’ structure, with a smoother surface indicating a well-optimized fabrication and replication process.

### Micro-vibration assisted MNs insertion and ISF extraction

#### MN applicator with arduino microcontroller

**Fig. 6:**
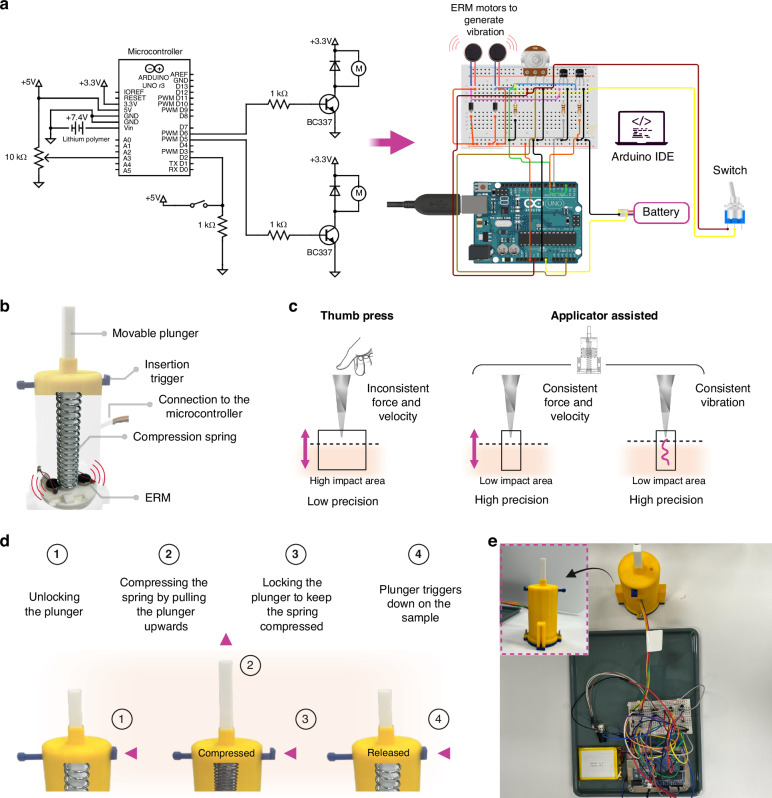
MN applicator and its operational mechanisms of the design and functionality. **a** Schematic diagram of the electronic control system for the applicator, showing the connections between the microcontroller (Arduino Uno) connected with two ERM motors using a potentiometer, two transistors, two resistors, two diodes, a switch, and a battery. The switch enables vibration at 50 Hz or 100 Hz frequencies when activated. **b** Illustration of the internal configuration of the plunger mechanism with two ERMs for providing vibration, highlighting the insertion trigger, connection to the microcontroller, and compression spring. **c** Comparative biomechanics of thumb press versus applicator-assisted MN insertion methods, highlighting the differences in applied force, insertion velocity, and precision. **d** Step-by-step operational mechanism of the applicator from unlocking the plunger to triggering it down onto the sample. **e** Photograph of the assembled applicator configuration with an inset showing a close-up view of the custom-built applicator

Our group has previously developed an MN applicator capable of generating vibrations at frequencies ranging from 50 to 250 Hz^40^. This study has integrated the applicator with the Arduino platform to enhance its precision and control. Overall design parameters such as height, width or main body of the applicator remained unchanged and consistent with the previous design. However, significant optimizations were made to the circuit connections. Initially, the applicator featured a single-vibration motor (ERM or LRA). In the modified version, two single ERM motors were attached parallel to each other on the plunger, controlled and synchronized using the Arduino Integrated Development Environment (IDE), as shown in Fig. 6a. This setup created symmetrical vibrations across the plunger head, which led to a more uniform distribution of the vibration pattern. In contrast, placing just one motor on the side may result in uneven vibrations, with stronger effects closer to the motor and weaker effects farther away. ERM motors were preferred for their energy efficiency and low cost, producing primarily transverse vibrations. These were programmed via the Arduino IDE to manage the operations of the motors and precisely control the vibration frequency (50 Hz and 100 Hz). This eliminated the need for manual tuning with a potentiometer knob, which can be imprecise and challenging to set accurately to the required frequencies (50 Hz or 100 Hz). This imprecision could lead to inconsistent outcomes, especially if the vibration frequency is not properly optimized.

The programming through the Arduino IDE enabled finer control than manual adjustments (potentiometer), enhancing the system’s overall functionality. Despite this, a potentiometer was still incorporated as a backup control method, allowing for manual adjustments of the voltage applied to the ERM motors when necessary. This ensures that manual control can still be available if the Arduino IDE is not accessible. Therefore, two transistors were introduced to amplify the signal to the required voltage level. Diodes were also added to protect the transistors from reverse voltage spikes and maintain circuit stability. Furthermore, resistors were used to limit the current flowing through the transistors and motors, ensuring safe and efficient operation. When the Arduino reads signals from the potentiometer, it converts them to voltage, which then controls the vibration of the two ERMs, translating electrical signals into physical vibration. Figure 6b shows the mechanical design and internal configuration of the MN applicator. The insertion trigger initiates the action, while the compression spring provides the necessary force for the plunger to push the MNs into the skin.

One of the fundamental challenges for swellable MNs is the consistent and precise skin penetration, which is influenced by various factors such as the applied force, the velocity of insertion, and the mechanical properties of the MNs^36^. While the thumb press (manual application) method is simple and accessible, it may lead to uneven penetration depths and inconsistent results. Moreover, manual insertion produces inconsistent force and velocity, resulting in unpredictable high or low impact with low precision. Observations from biological systems, such as mosquitoes and honeybees, reveal that their proboscis insertion happens remarkably quickly^41,42^. Moreover, mosquitoes use a combination of mechanical action and micro-vibrations to penetrate the skin efficiently with minimal discomfort^43^. This highlights the natural effectiveness of velocity and vibration in efficiently penetrating skin surfaces. Therefore, the applicator was developed to address these challenges by offering significant benefits for skin insertion with minimal invasiveness. With its ability to control the impact applied, the applicator can provide greater precision in the MN application and ISF extraction effectiveness. Figure 6c highlights the skin biomechanics of applicator-assisted MN application compared to manual insertion.

The step-by-step operational mechanism of the applicator, which has a velocity of 4.5 m/s and vibrations of 50 and 100 Hz, is shown in Fig. 6d. The mechanism starts by unlocking the plunger, pulling it upward, compressing the spring and storing potential energy. The plunger is then locked to maintain this compression. When triggered, the stored energy in the spring is released, driving the MNs into the skin sample with controlled force and precision. Figure 6e shows the actual physical setup of the applicator device connected to the electronic component for the MN insertion test.

#### MNs insertion, ISF extraction, and glucose recovery

An initial parafilm test was performed to investigate the impact of micro-vibration on skin penetration. It involved manual insertion and applying vibration frequency at 50 Hz and 100 Hz on a 1.1 mm skin model comprising 8 layers of parafilm. The first layer of the parafilm is critical as it represents the initial interaction of the MNs with the SC^44^. Testing on the first layer provides the most relevant information on how the MNs would perform on the skin.

**Fig. 7:**
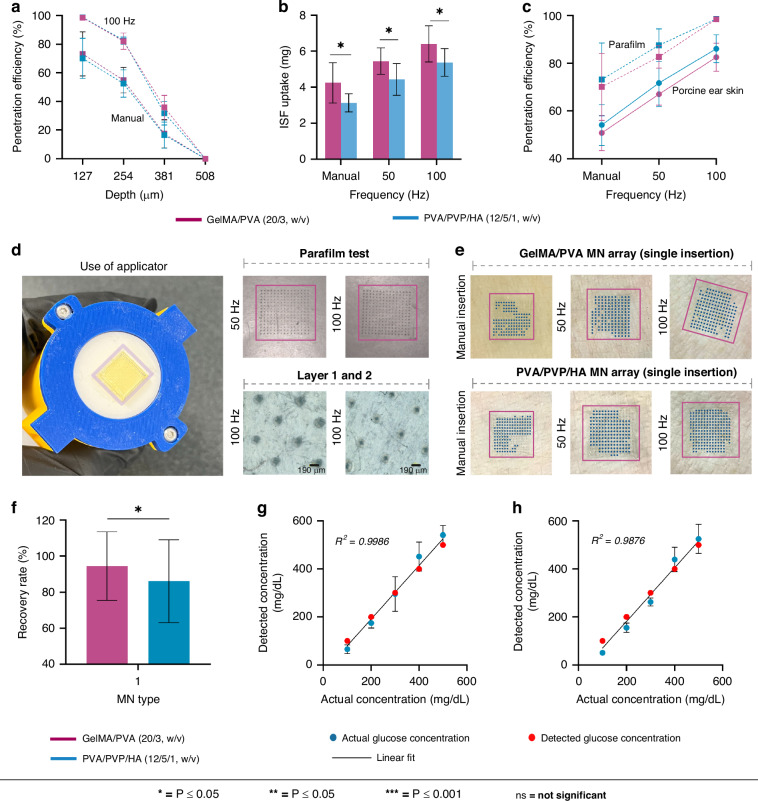
Assessment of the spiral MN’s insertion and extraction capabilities. **a** Penetration efficiency (%) at different parafilm depths (127, 254, 381, and 508 µm) under varying frequencies (manual and 100 Hz). **b** The volume of ISF uptake (mg) from porcine ear skin at different frequencies (manual, 50 Hz, and 100 Hz). **c** Comparison of the penetration efficiency (%) of the MNs across different frequencies (manual, 50 Hz, and 100 Hz) between parafilm and porcine ear skin. **d** MN array attached to the applicator’s plunger head using a double-sided tape for the insertion tests on parafilm shows the impact of 50 Hz and 100 Hz frequencies on the uniformity in first and second parafilm layers for GelMA/PVA (20/3, w/v) MNs. **e** Micro-holes created by the MNs visible on the porcine ear skin at different frequencies (manual, 50 Hz, and 100 Hz) **f** Bar diagram of the glucose recovery rate (%) for both MN types. **g** The detected glucose concentrations by the GelMA/PVA (20/3, w/v) MNs in agarose hydrogel (blue dots) versus the actual glucose concentration (red dots). **h** The detected glucose concentrations by the PVA/PVP/HA (12/5/1, w/v) MNs in agarose hydrogel (blue dots) versus the actual glucose concentration (red dots)

The percentage of spiral MNs in an array which penetrated the skin model without vibration for the first layer was 70 ± 14% and 73 ± 0.39% for the GelMA/PVA (20/3, w/v) and PVA/PVP/HA (12/5/1, w/v) respectively. When vibration was introduced (50 Hz), GelMA/PVA (20/3, w/v) MNs exhibited parafilm micro-hole distributions of 82 ± 4.79%, increasing the penetration efficiency on the first layer by 17.14%. Similarly, for PVA/PVP/HA (12/5/1, w/v) MNs, the micro-hole percentages for the first layer were 87 ± 6.86%, which is a 19.17% increase from the manual insertion. Results further showed that increasing the frequency from 50 Hz to 100 Hz significantly increased the penetration efficiency to 98 ± 0.5% and 98 ± 0.3% for GelMA/PVA (20/3, w/v) and PVA/PVP/HA (12/5/1, w/v), respectively. Although both types of MNs could penetrate multiple layers of parafilm, the number of holes decreased sequentially within each layer. It was also noted that the diameter of the holes in the parafilm on the first layer is larger than those on the second and third layers. Maximum penetration depth calculated for both MN types reached 381 µm within parafilm, Fig. 7a. Considering the average thickness of the SC is 10–30 µm^45^, the results demonstrated that the applicator with vibration enhanced the penetration efficiency and produced more micro-holes on the skin model, which is a critical improvement from manual insertion and vital for the successful extraction of ISF. Because of the 4.5 m/s approach speed of the applicator, penetration to the full depth of 381 µm occurs without multiple transverse vibrations of the MNs. However, during the post-insertion sprung contact time of a few seconds immediately following insertion, hundreds of vibrations occur (before the vibrations are turned off), which widen each of the MN pores with significant improvement of penetration efficiency and eventual fluid uptake.

Although the parafilm test provides valuable insights for analyzing the skin penetration capabilities of the MNs before testing on biological samples (porcine ear skin), It has been reported to demonstrate higher penetration depths than those observed in porcine skin^46^. Therefore, the insertion test of MNs was further studied on porcine ear skin to understand the difference between the use of vibration-enhanced and manual application (using thumb pressure). MN arrays were applied manually and with the custom-made applicator (50 Hz and 100 Hz) at a speed of 4.5 m/s from a specific distance on the porcine ear skin. For GelMA/PVA (20/3, w/v), penetration efficiency on the porcine ear skin with manual insertion and the applicator with vibration enhancement at 50 Hz and 100 Hz were 50 ± 7.4%, 67 ± 4.6%, and 82 ± 5.9%, respectively. Result indicated a decrease of 28.57% (manual), 18.29% (50 Hz), and 16.32% (100 Hz) in penetration efficiency compared to the tests conducted on Parafilm. Similarly, For PVA/PVP/HA (12/5/1, w/v) MNs, higher frequencies showed improved penetration rates with 54 ± 8.55% for manual insertion, 71 ± 9.92% at 50 Hz), and 86 ± 5.84% at 100 Hz. However, there was also a decrease in penetration efficiency of 26.02% for manual insertion, 18.39% at 50 Hz, and 12.24% at 100 Hz compared to Parafilm test results.

After MN arrays were manually applied for 5 min on the porcine ear skin, ISF uptake for GelMA/PVA (20/3, w/v) was only 4.24 ± 1.1 mg. However, using the applicator, the ISF uptake of GelMA/PVA increased to 5.45 ± 0.7 mg and 6.41 ± 1.0 mg for 50 Hz and 100 Hz, respectively. For PVA/PVP/HA (12/5/1, w/v), thumb-pressed MN arrays extracted 3.13 ± 0.5 mg of ISF, while using the vibration-enhanced applicator improved the extraction to 4.43 ± 0.8 mg and 5.38 ± 0.7 mg for 50 Hz and 100 Hz respectively, Fig. 7b. As expected, the penetration efficiency at different vibration frequencies (manual, 50 Hz, 100 Hz) was higher in parafilm compared to porcine ear skin, as shown in Fig. 7c. The correlation between penetration efficiency and the volume of ISF extracted is apparent as the higher penetration efficiency at (50 Hz and 100 Hz) led to a higher volume of ISF uptake. The result indicated that at a frequency of 100 Hz, MNs exhibited a significantly higher penetration rate, creating a greater number of viable micro-holes and simultaneously widening each micropore in the porcine ear skin compared to parafilm, Fig. 7d, e. The reduced efficiency with manual pressing compared to applicator-assisted operation could be attributed to uneven force distribution across the MN array. Human fingers are less precise in maintaining consistent pressure, leading to variability in penetration efficiency and depth^47^. The force distribution mechanism across skin structure during MN insertion, manual versus applicator-assisted, is shown in Supplementary Fig. S4.

The velocity of MN insertion is also a critical factor influencing skin resistance during penetration. Studies have shown that increasing the insertion velocity reduces the force required to penetrate the skin^48^. This effect is particularly significant in viscoelastic materials like skin, where higher velocities cause greater deformation, thereby lowering the skin’s fracture strength and enhancing skin penetration^49,50^. This is an important aspect for swellable MNs, which typically have lower mechanical strength than other types due to their swellability. Moreover, the dynamic nature of vibration allows MNs to overcome the skin’s natural viscoelastic resistance more effectively than a static application of force. The combination of velocity (4.5 m/s) and vibration (50–100 Hz) enables the swellable MNs to penetrate deeply into the skin with minimal push force. While manual pressing might theoretically apply more force, it also lacks the micropore-widening effect, leading to lower fluid uptake efficiency. Moreover, excessive pressure could potentially damage the MNs or cause discomfort. Therefore, a vibration frequency of 100 Hz achieved optimal outcomes for the test on porcine ear skin, enhancing both penetration efficiency and micropore widening, which together lead to improved ISF extraction.

Nonetheless, due to the swelling nature of the hydrogel MNs, their structure weakens upon penetrating the SC, making them less capable of enduring continuous vibration. The ability of MNs to withstand vibration-enhanced penetration of the SC is also related to mechanical strength of the MNs. Higher frequencies (>100 Hz) demand greater mechanical strength from the MNs in transverse motion while inserted in the SC for several seconds after insertion. Therefore, vibrations were discontinued during the ISF extraction process to avoid the dissolution of the MNs. The volume obtained by manual insertion is also variable and prone to inconsistencies due to randomly applied force, depth, and angle of penetration. This lack of precision can lead to uneven penetration depths and varying degrees of skin disruption, resulting in unreliable outcomes and suboptimal ISF collection. The applicator ensures that a consistent force and insertion speed (4.5 m/s) is used and reduces variability between repetitive applications. This uniformity is crucial for achieving reliable and reproducible results in ISF extraction.

To assess the ability of MNs to recover biomarkers, glucose was chosen as the target biomolecule within the agarose gel matrix. The MN arrays were applied to the agarose matrix for 5 min, after which they were removed and dissolved at 60 °C to recover the glucose. The results suggested that the volume of ISF extracted can influence the recovery rate of glucose, as shown in Fig. 7f. For example, recovery rate of GelMA/PVA (20/3, w/v) and PVA/PVP/HA (12/5/1, w/v) MNs were ~94.48% and ~86.08%, respectively showed that the increase in volume of ISF uptake of GelMA/PVA (20/3, w/v) led to a higher glucose recovery rate compared to PVA/PVP/HA (12/5/1, w/v) MNs. Small volumes of ISF could contain significantly fewer glucose molecules, leading to increased variability and a higher likelihood of measurement errors. This can make it challenging to achieve accurate glucose readings, as the reduced number of glucose molecules may fall below the sensitivity threshold of the glucose detection assay kit. The accuracy of our results is supported by the fact that the glucose detection kit used is highly sensitive, which comfortably detects the induced glucose concentrations of 100 mg/dL to 500 mg/dL. Additionally, the extracted ISF volumes exceeded 5 μL for all MN types, which ensured a higher concentration of glucose within the ISF for the detection. A study has also shown that larger volumes of ISF provide more stable and accurate glucose detection, as they reduce the impact of local variations and potential contamination^51^. The correlations showed that the detected glucose concentrations (blue dots) closely aligned with the actual concentrations (red dots) across the entire range, with the data points signifying a high level of accuracy (*R*^2^ = 0.9986 for GelMA/PVA (20/3, w/v), *R*^2^ = 0.9876 for PVA/PVP/HA (12/5/1, w/v).

## Conclusion

Considering the findings presented, our research addresses some of the key issues and research gaps identified in previous literature concerning the successful extraction of ISF using MNs. It has been highlighted that two of the critical challenges are the sample volume and extraction time^30^. While there are currently no set standards for ISF volume and extraction time, it is essential to recognize the potential risks associated with low ISF volume, as it may result in obtaining insufficient or no biomarkers. Additionally, maintaining a relatively short extraction time is essential for preserving the integrity of hydrogel MNs and ensuring successful ISF extraction. This research addressed these critical challenges by introducing innovative design, novel approaches, material optimization, and developing an efficient ISF extraction process using a custom-made applicator. These connections underscore the significance of the findings in contributing to advancements in MN technology for improved, accurate and practical diagnostics and therapeutic responsiveness.

The performance of MNs in extracting ISF is influenced by several factors, including their design, material, swelling ability, mechanical characteristics, and insertion technique. Therefore, one of the objectives of this study was to investigate using only GelMA as the MN material and assess its ability to extract a higher volume of ISF within a short timeframe (>5 min). Previous research suggests that while GelMA and GelMA-based MNs can extract ISF, volumes typically remain below 5 µL, and extraction times often exceed 10 min. Therefore, we introduced PVA as an additional hydrophilic polymer within the GelMA matrix to improve the extraction volume of ISF. The introduction of PVA aimed to explore and enhance the swelling properties of MNs. A comprehensive analysis was conducted by varying the PVA concentrations at 3% (w/v) and 5% (w/v). The objective was to investigate how these different PVA concentration combinations would impact the swelling behavior of the MNs and the overall performance in ISF extraction. The results showed that small amount of PVA (3%, w/v) within the GelMA substantially enhances the swelling performance of MNs, which has been validated through testing on porcine skin.

This study also addressed the limitations of research studies relying solely on PVA, PVP, or similar hydrogel compositions for ISF extraction. Thus, HA, which has exceptional water retention capabilities to enhance extraction, was utilized. Three different concentrations of HA (1%, 2%, 3%, w/v) were combined with PVA/PVP. The results revealed that a small amount of HA (1%, w/v) incorporated with PVA/PVP acted as an enhancer and was adequate to uptake a higher volume of ISF within 5 min.

Relatively large MN arrays measuring 12 cm × 12 cm, housing 225 spiral MNs were created. The resulting MN configurations, featuring intact tips and complex spiral structures, demonstrated great precision and mechanical stability. However, due to the intricate geometry and size, the demolding process is more challenging for the larger MN array than a smaller equivalent. Addressing this, we optimized the design and demolding procedures to overcome initial inconsistencies, leading to enhanced and more efficient fabrication results. In addition, a crucial objective was the development of a flat and robust MN base surface. A well-optimized base is essential for even pressure distribution during the application of successful MN insertion. Consequently, the base layer was fine-tuned using PVP for seamless and rapid integration with the MNs.

The skin penetration process is critical in successful ISF extraction; hence, a novel approach was introduced. MNs were applied through a custom-made applicator with integrated micro-vibration motors controlled by an Arduino IDE. Transverse vibration was applied at two different frequencies, 50 Hz and 100 Hz. Introducing micro-vibration via the custom-made applicator proved to be significantly impactful, enabling excellent ISF extraction in just 5 min, a stark contrast to other methods. Moreover, to extract the glucose as the target biomarker from the MNs in vitro, a mild heating process was employed and validated by comparing the measured glucose levels with the actual ones. The close correlation between the two suggests that the ability of the MNs to extract ISF for glucose level detection holds great promise for accuracy.

## Experimental section

### Materials

Polyvinyl alcohol with two molecular weights (PVA, Mw 89 kDa and Mw 30 kDa, fully hydrolyzed), polyvinylpyrrolidone (PVP, Mw 40 kDa, fully hydrolyzed), sodium hyaluronate (HA, Mw 300 kDa), d-glucose, agarose (low EEO), glutaraldehyde (grade II, 25% in water), methacrylic anhydride (MA, purity 94%), glucose oxidase (GO) assay kit, 2-hydroxy-4′-(2-hydroxyethoxy)-2-methylpropiophenone (Irgacure-2959), Dulbecco’s phosphate-buffered saline (PBS, pH 7.1–7.5) were purchased from Sigma-Aldrich (St. Louis, MO, USA). Sylgard 184 silicone elastomer was purchased from Dow Corning (Midland, MI, USA). Artificial interstitial fluid (pH 7.4) was purchased from Biochemazone (Alberta, Canada). All electronic components were purchased from Core Electronics (NSW, Australia).

All materials were obtained of the highest quality and used without further purification. Porcine cadaver ears were freshly obtained from a local butcher shop (Brisbane, Australia).

### Master MN array printing and PDMS molds preparation

Master MN array was printed using projection micro stereolithography (PμSL) by a BMF microArch^®^ S240 ultrahigh resolution 3D printer from Boston Micro Fabrication (BMF, Maynard, MA, USA). MN molds were created by thermal curing a PDMS solution using the master MN array as a template. To prepare the PDMS solution, the silicone elastomer base and silicone curing agent (SYLGARD 184 Silicone Elastomer Kit, Dow Corning, Midland, MI, USA) were thoroughly mixed at a ratio of 10:1 (base: agent) and centrifuged (Kurabo KK-50S, Kurabo Electronics, OSK, Japan) for 4 min to yield a homogenous mixture. The mixture was poured onto the master MN array, placed in a petri dish, and cured at 90 °C for 2 h in a standard laboratory oven (Labwit Scientific, ZXRD-A5055, Australia). Once the PDMS had fully cured, it was gently peeled off from the surface of the PDMS mold to be used for MN replication.

### Synthesis of GelMA

GelMA was prepared following a protocol reported previously^1^. Briefly, 10 g of gelatin was added to 100 ml of PBS at 50 °C while stirring continuously until fully dissolved. Then, 10 ml of methacrylic anhydride (MA) was slowly introduced to the solution and stirring continued for an additional 3 h at 50 °C to facilitate the reaction. Five Hundred millilitres of warm (40 °C) PBS was added to the solution to reduce the concentration of unreacted reagents. The solution was transferred into a dialysis bag (12–14 kDa membrane) and dialyzed in ultrapure water for 7 days to remove unreacted MA and small molecules. After dialysis, the clear solution was collected in a reagent bottle and placed in a freezer for 48 h at −20 °C. The frozen solution was transferred into several beakers and freeze-dried (VaCo 5-D, Zirbus Technology GmbH, Harz, Germany) for 5 days at −80 °C. This resulted in a white, porous GelMA prepolymer foam with a yield of 5.8 g from the initial 10 g. The prepolymer was stored at 4 °C before use.

### Degree of substitution of GelMA

The methacrylamide groups in GelMA were quantified at 40 °C by a high-resolution ^1^H NMR spectrometer (Bruker Avance III 500 MHz, Bremen, Germany). GelMA and Gelatin samples were prepared by dissolving 40 mg GelMA or gelatine in 0.7 mL D_2_O. The degree of substitution (DS) of methacrylamide on gelatine (Supplementary Fig. S5) was calculated using the integrals of lysin methylene protons of GelMA and gelatine using the following equation:

1 $${DS}\left( \% \right)=1-\frac{\int {of\; lysine\; methylene\; proton\; of\; GelMA}}{\int {of\; lysine\; methylene\; proton\; of\; gelatin}}\times 100$$

### Fabrication of GelMA and GelMA/PVA MNs

Three different solutions were prepared in PBS for MN arrays replication: GelMA (20%, w/v), GelMA/PVA (20/3, w/v) and GelMA/PVA (20/5, w/v). The porous GelMA prepolymer was initially added to PBS at 50 °C until fully dissolved. The aqueous GelMA solution was then mixed with the photoinitiator Irgacure (0.5%, w/v) at 50 °C to prepare the GelMA solution. Separately, PVA (Mw 30 kDa) was dissolved in PBS at two concentrations (3 and 5%, w/v) under continuous stirring at 70 °C overnight. Subsequently, GelMA (20%, w/v) was added to each homogeneous PVA solution at 50 °C and stirred for 4 h. Irgacure (0.5%, w/v) was then added, and the mixture was placed in an ultrasonic bath at 40 °C for 15 min. The prepared solutions were cast into the MN molds and centrifuged at 4000 rpm for 10 min to fill the MN molds cavities. MN molds were then exposed to UV light (360–480 nm) for 250 s. The cross-linked molds were dried in the dark at 37 °C before being peeled off for further use.

### Fabrication of PVA/PVP/HA MNs

PVA (Mw 89 kDa, 12%, w/v) was dissolved at 95 °C for 2 h in PBS under continuous stirring. PVP (Mw 40 kDa, 5% w/v) was also dissolved using the same method, while different concentrations of HA (Mw 300 kDa) were prepared separately at 1%, 2%, and 3%, w/v. PVP (Mw 40 kDa, 30%, w/v) was dissolved in Milli-Q water at 85 °C for an hour to create the base solution. Then, PVA/PVP/HA solutions were set by accurately combining the pre-made PVA, PVP, and HA solutions. This was followed by adding a small amount of crosslinker glutaraldehyde to the reaction mixture at 95 °C for 1 h. The PVA/PVP/HA solution was pipetted into the MN molds and centrifuged at 4000 rpm for 10 min to ensure complete penetration into the molds cavities. To form a robust MN base, the molds were lightly overfilled twice periodically with the base solution PVP (Mw 40 kDa, 30%). The filled molds were dried at 37 °C f or 36 h before being peeled off for further use.

### Arduino-based custom-made applicator

The custom-made applicator was printed using a 3D printer (Teirtime ×5, Teirtime Corporation, USA) with polylactic acid filament with a similar geometry described in our group’s previous work^30^. The updated version of the applicator is equipped with the Arduino Uno microcontroller to control the system, which includes ERM vibration motors, a diode rectifier, a lithium polymer battery, a potentiometer, a transistor, and a resistor. The frequency range was directly adjusted between 50 and 100 Hz using the Arduino IDE.

The knob controlling the speed of the groove plunger was set to 4.5 m/s with an impact energy of 215.46 mJ. A push-button mechanism triggered the plunger head (with attached MN arrays) to impact the sample (Parafilm and porcine ear skin) while the vibration (50 Hz or 100 Hz) was introduced using the Arduino IDE. The impact velocity of the prototype was validated using two techniques: high-speed camera analysis and theoretical modeling, as determined in our prior study^30^.

### Swelling ratios of the MNs in PBS and artificial ISF

The swelling ratios of MNs were assessed by measuring changes in mass before and after being submerged in both PBS and artificial ISF solution. MN array samples were submerged in PBS and artificial ISF at 37 °C to determine the difference between wet weight (*W*_*w*_) and dry weight (*W*_*d*_) at different time points (1–10 min). The objective was to observe the early stages (>10 min) of swelling ratios and analyze the trend over time by monitoring the mass changes at relatively short intervals (1 min). The swelling ratio of the MNs was calculated using the following equation:

2 $${Swelling\; ratio}( \% )=\left[\frac{{W}_{w}-{W}_{d}}{{W}_{d}}\right]\times 100$$

### Determination of mechanical properties

Mechanical properties of the MNs were assessed by compression tests using a low-force UTM (Instron 3343, Instron, MA, USA). For each test, a MN array (15 × 15) was placed needle-side up on the fixed plate of the UTM using a double-sided tape and compressed at a rate of 0.5 mm/min up to a maximum downward height of 0.5 mm with a 50 N static load cell. The primary failure mode recorder was buckling with no significant torsional effects or additional twisting at failure. Force vs displacement was recorded to calculate the compressive modulus (*E*) from the strain (*σ*) and stress (*ϵ*) curve with the following equations:

3 $${Stress}\left(\sigma \right)=\frac{F}{A}$$

4 $${Strain}(\epsilon )=V(t-{t}_{0})$$

5 $${Compressive\; modulus}(E)=\frac{\sigma }{\epsilon }$$

Where *F* is the applied compressive force (N), *A* is the cross-sectional area of the MN array where the compressive force is applied (mm²), *V* is the set rate of the load cell of the UTM, *t* is time of elastic deformation and *t*_*0*_ is an approximate time when the load cell first reaches the MNs.

### Morphology characterization

Images of the spiral MN arrays (master and replicas) were captured using a digital microscope (DSX1000, Olympus, Japan) at different magnifications (×20, ×40). The imaging was conducted with adjustable tilt angles ranging from 0° to 45°.

The spiral morphology of the MNs was assessed using a scanning electron microscope (SEM, JSM-7001F, JEOL, Japan). The MNs were sputter-coated with a 12 nm thick layer of platinum to prevent charging. The sample was then imaged in the field emission SEM at an accelerating voltage of 10 kV in high vacuum mode (HighVac) with a magnification level of ×50. MN arrays were carefully de-molded just before the imaging to prevent any potential damage.

### Surface characterizations

Atomic force microscopy (AFM, Multimode 8-HR Nanoscope System, Bruker, CA, USA) was used in ScanAsyst mode to map the surface topography of the MNs. Silicon cantilevers (ScanAsyst Air, Bruker AFM Probes, CA, USA) featuring spring constants within the range of 20–80 N/m and resonance frequencies between 45–95 kHz were used for the measurements. For sample preparation, GelMA/PVA (20/3, w/v) and PVA/PVP/HA (12/5/1, w/v) solutions were individually deposited into pre-made circular blocks (10 mm diameter) and subsequently dried for 36 h at 37 °C prior to testing. The scanning parameters were configured with a scan size of 500 nm at 2 Hz. The post-image analysis was conducted offline using the NanoScope Analysis software (Bruker, CA, USA).

### Insertion test and extraction of ISF ex vivo

A commercially available Parafilm sheet was stacked into eight layers (127 µm per layer, combined ~1 mm) to simulate human skin for the initial insertion test. MN arrays were applied to the simulated skin setup both manually and using the custom-made applicator at frequencies of 50 Hz and 100 Hz. After insertion, the micro-vibration process was stopped after a few seconds during which micropore widening occurred, after which the MN arrays were left in place for 30 s before being removed. The holes in the Parafilm layers were then examined under a microscope (Carl Zeiss Axio, Jena, Germany) to assess the efficiency of insertion.

Freshly slaughtered porcine ear skin was sourced from a local butcher (Brisbane, Australia) and stored in aluminum foil at −20 °C until used. Before MN array insertion, the skin was cleaned with ultra-pure water and thawed in PBS for 2 h at 37 °C. The thickness of the sample was measured at 3.2 ± 0.49 mm. Initially, the MN array was directly applied to the porcine ear skin without using the custom-made applicator. Subsequently, the MN arrays were inserted using the applicator at a speed of 4.5 m/s, with vibration applied solely during the insertion process and a few seconds afterwards at 50 Hz and 100 Hz frequencies. However, no vibration was applied during the ISF extraction phase. The array was left in place for 5 min before being removed. The obtained ISF volume was determined by subtracting the initial weight of the array (*W*_*d*_) from the weight measured after extraction (*W*_*w*_). Trypan blue (0.4%, w/v) was used to identify and stain the skin and incubated for 5 min to capture optical images.

### Detection of glucose concentration in vitro

A 3% (w/v) agarose hydrogel was prepared with varying glucose concentrations: 100, 200, 300, 400, and 500 mg/dL. To detect the glucose concentration by MN arrays, the array’s dry weight (*W*_*d*_) and wet weight (*W*_*w*_) were measured after being inserted into the agarose gel containing different glucose concentrations for 10 min in each test. After insertion, the MNs were carefully removed from the array and placed in centrifuge tubes containing 1 ml of PBS (pH 7.4). The tubes were heated at 60 °C for 15 min to release glucose. Absorbance was measured at 540 nm by a UV–vis spectrophotometer (Shimadzu, UVmini-1240, Japan) and quantified using a commercial glucose assay kit. Glucose concentration detected (*C*_*d*_) by the MN arrays were determined using the following formula:

6 $${C}_{d}=\frac{C\times V}{({W}_{w}-{W}_{d})\times \rho }$$

Where *C* = glucose concentration from the standard curve using the assay kit, *V* = volume of the PBS added into the centrifuge tube (1 mL), *W*_*w*_ = wet mass of the MN array, *W*_*d*_ = dry mass of the MN array, *ρ* = density of the glucose extracted by the MNs (~ 1.0 g/mL).

### Statistical analysis

The statistical analysis of the data was performed using one-way ANOVA, with significance levels denoted by asterisks: * for *p* ≤ 0.05, ** for *p* ≤ 0.01, and *** for *p* ≤ 0.001. Origin Pro 2022 and Python were used for all statistical calculations. The data are presented as the mean ± standard deviation from five independent experiments (*n* = 5).

## Supplementary information

Supplementary information
