# Supplementary material for: Micro-vibration assisted dual-layer spiral microneedles to rapidly extract dermal interstitial fluid for minimally invasive detection of glucose
Source: Microsyst Nanoeng. 2025 Jan 8;11:3. doi: 10.1038/s41378-024-00850-x (PMC11706973; doi:10.1038/s41378-024-00850-x)
Supplement: Supplementary file 1 — Supplementary information [file 41378_2024_850_MOESM1_ESM.docx]

Supplementary Information

Micro-vibration Assisted Dual-Layer Spiral Microneedles to Rapidly Extract Dermal Interstitial Fluid for Minimally Invasive Detection of Glucose

Khaled Mohammed Saifullah^1,2^, Asim Mushtaq^2^, Pouria Azarikhah^1,2^, Philip D Prewett^3,4^, Graham J Davies^5,6^, Zahra Faraji Rad*^1,2^

^1^ School of Engineering, University of Southern Queensland, Queensland 4300, Australia

^2^ Centre for Future Materials, Institute for Advanced Engineering and Space Sciences, University of Southern Queensland, Queensland, Australia

^3^ School of Engineering, University of Birmingham, Birmingham B15 2TT, United Kingdom

^4^ Oxacus Ltd, Dorchester-on-Thames, OX10 7HN, United Kingdom

^5^ Faculty of Engineering, UNSW Australia, NSW 2052, Australia

^6^ College of Engineering & Physical Sciences, School of Engineering, University of Birmingham, Birmingham, B15 2TT, United Kingdom


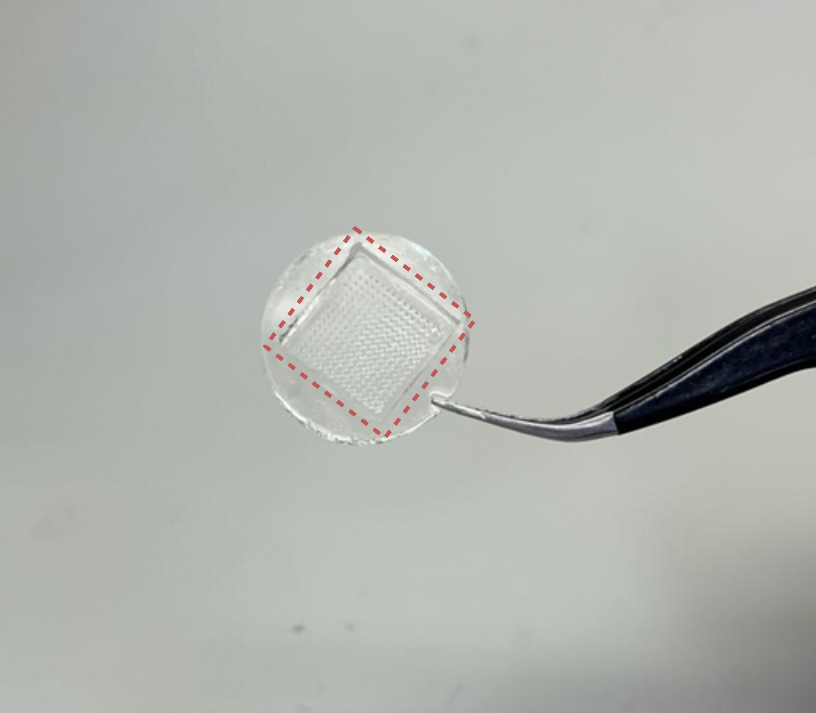


**Figure S1.** A close-up view of the circular mold containing a square array (highlighted in red) of MN cavities. The mold is approximately 20 mm in diameter.


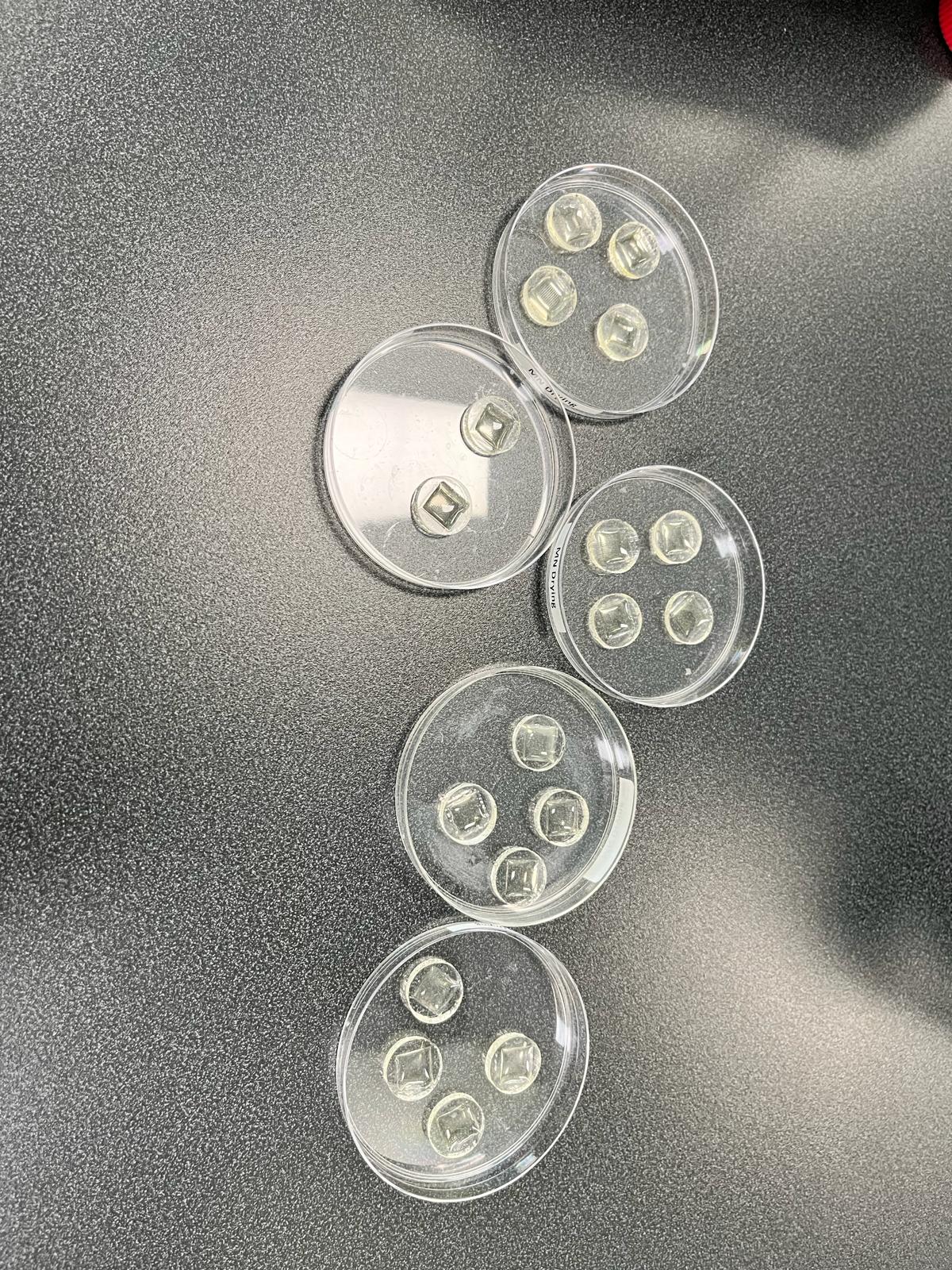


**Figure S2.** Petri dishes containing MN molds are filled with MN solution to be incubated for optimal drying.


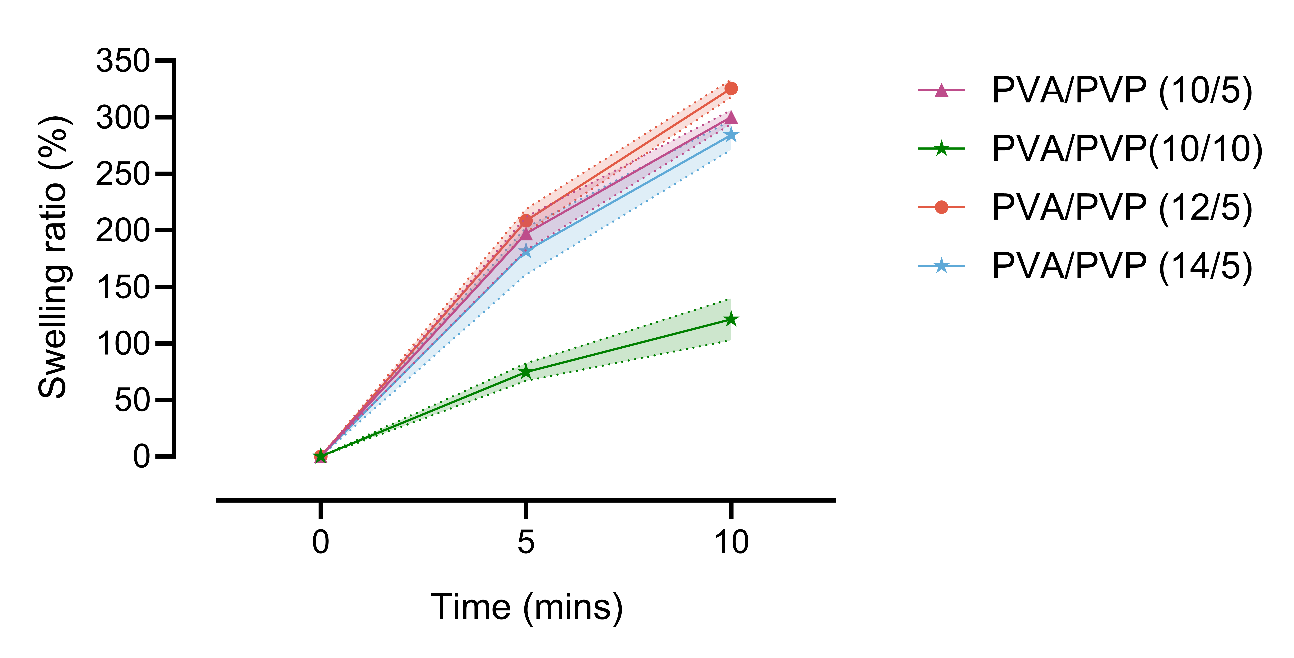


**Figure S3.** Swelling ratio of MN arrays at different PVA/PVP compositions at 5 minutes and 10 minutes.

**Table S1.** Comparative swelling performance of GelMA and PVA/PVP/HA hydrogels in PBS and Artificial ISF at 37 °C.

| Material | Condition | 5 Minutes  (Mean ± SD) | 10 Minutes  (Mean ± SD) |
| --- | --- | --- | --- |
| GelMA (20%, w/v) | Artificial ISF | 160 ± 27.13 | 230 ± 20.87 |
|  | PBS | 137 ± 9.55 | 213 ± 6.93 |
| GelMA/PVA (20/3, w/v) | Artificial ISF | 449 ± 58.64 | 560 ± 79.61 |
|  | PBS | 438 ± 58.52 | 555 ± 89.87 |
| GelMA/PVA (20/5, w/v) | Artificial ISF | 292 ± 24.95 | 432 ± 35 |
|  | PBS | 281 ± 14.82 | 418 ± 20.15 |
| PVA/PVP/HA (12/5/1, w/v) | Artificial ISF | 254 ± 17.60 | 370 ± 18.51 |
|  | PBS | 247 ± 34.10 | 358 ± 45.45 |
| PVA/PVP/HA (12/5/2, w/v) | Artificial ISF | 198 ± 40.87 | 286 ± 48.09 |
|  | PBS | 192 ± 28.90 | 273 ± 52.88 |
| PVA/PVP/HA (12/5/3, w/v) | Artificial ISF | 144 ± 21.77 | 239 ± 21.30 |
|  | PBS | 146 ± 7.60 | 206 ± 10.73 |


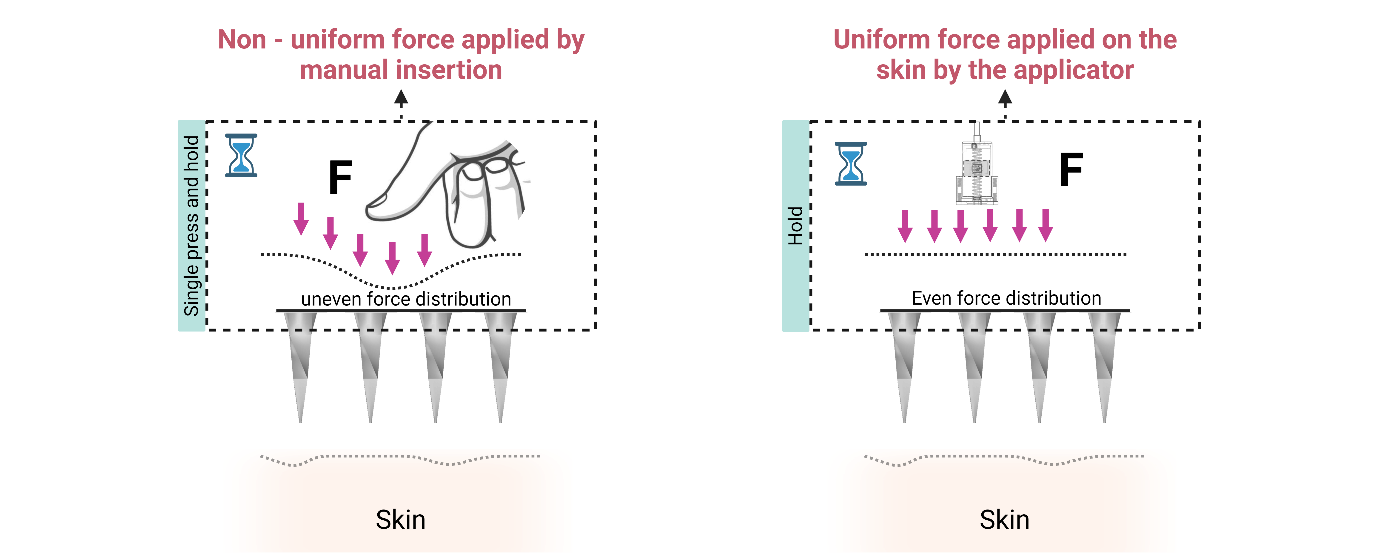


**Figure S4.** The difference between the uneven force distribution using manual insertion of MNs (left) and the even force distribution achieved by using the applicator system (right).


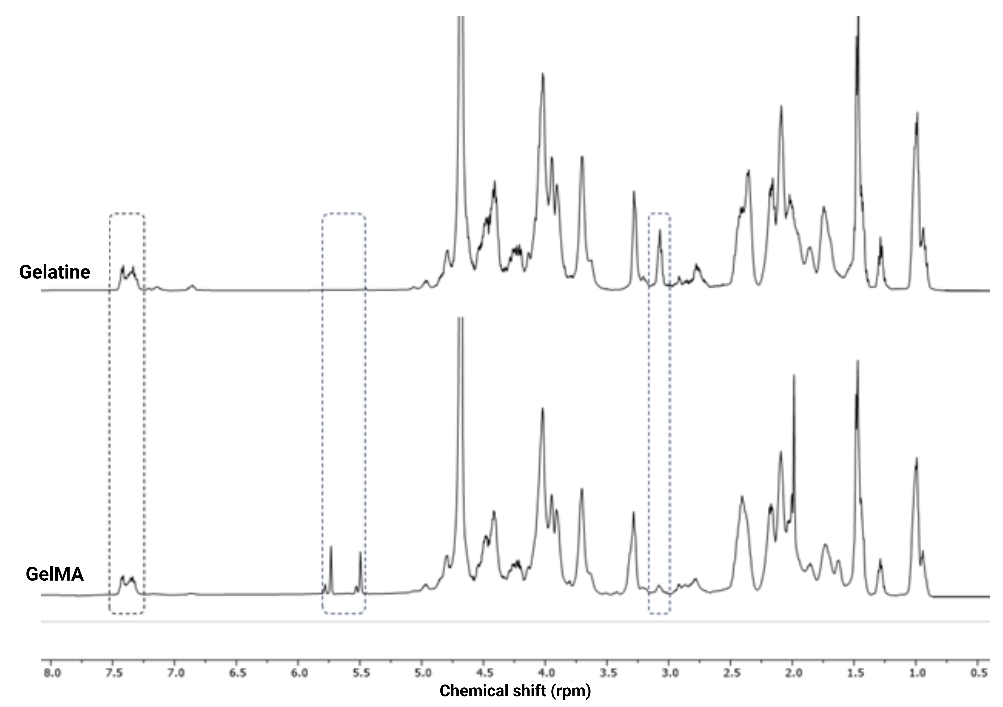


**Figure S5.** ^1^H NMR spectra of gelatine and GelMA. The functionalization peaks are highlighted at 3.06 ppm and 5.4 – 5.8 ppm. The successful conjugation of methacrylamide groups was confirmed with the appearance of new peaks at 5.4 – 5.8 ppm and 1.9 ppm corresponding to the methacryloyl groups of GelMA. The significant decrease of free lysine signal at 3.06 ppm in GelMA compared to gelatine also confirmed the successful conjugation.
